# Transcatheter Aortic Valve Implantation in Bicuspid Aortic Valve with Aortic Stenosis: a Meta-Analysis and Trial Sequential Analysis

**DOI:** 10.21470/1678-9741-2020-0146

**Published:** 2022

**Authors:** Jeffrey Shi Kai Chan, Sukhdeep Singh, Peter Eriksen, Lok Him Tsui, Amer Harky, for the Cardiovascular Evidence Review Collaboration (CvERC)

**Affiliations:** 1 Division of Cardiology, Department of Medicine and Therapeutics, Prince of Wales Hospital, Shatin, New Territories, Hong Kong.; 2 Faculty of Medicine, The Chinese University of Hong Kong, Shatin, New Territories, Hong Kong.; 3 School of Medicine, University of Liverpool, Liverpool, United Kingdom.; 4 Li Ka Shing Faculty of Medicine, The University of Hong Kong, Hong Kong.; 5 Department of Cardiothoracic Surgery, Liverpool Heart and Chest Hospital, United Kingdom.; 6 Liverpool Centre for Cardiovascular Science, University of Liverpool and Liverpool Heart and Chest Hospital, United Kingdom.

**Keywords:** Valvular Heart Disease, Bicuspid Aortic Valve, Transcatheter Aortic Valve Implantation, Meta-Analysis

## Abstract

**Objectives:**

Bicuspid aortic valve (BAV) is an important aetiology of aortic stenosis and the use of transcatheter aortic valve implantation (TAVI) has not been fully explored in this cohort. This systematic review and meta-analysis compared the outcomes of TAVI in stenotic BAV against tricuspid aortic valve (TAV).

**Methods:**

An electronic literature search was performed in PubMed, MEDLINE, EMBASE, and Scopus to identify all studies comparing TAVI in stenotic BAV *versus* TAV. Only studies comparing TAVI in BAV *versus* TAV were included, without any limit on the study date. Primary endpoints were 30-day and 1-year mortality, while secondary endpoints were postoperative rates of stroke, acute kidney injury (AKI), and permanent pacemaker (PPM) requirement. A trial sequential analysis (TSA) was performed for all endpoints to understand their significance.

**Results:**

Thirteen studies met the inclusion criteria (917 BAV and 3079 TAV patients). The BAV cohort was younger (76.8±7.43 years *vs*. 78.5±7.12 years, *P*=0.02), had a higher trans-aortic valve gradient (*P*=0.02), and larger ascending aortic diameters (*P*<0.0001). No significant difference was shown for primary (30-day mortality [*P*=0.45] and 1-year mortality [*P*=0.41]) and secondary endpoints (postoperative stroke [*P*=0.49], AKI [*P*=0.14], and PPM requirement [*P*=0.86]). The BAV group had a higher rate of significant postoperative aortic regurgitation (*P*=0.002). TSA showed that there was sufficient evidence to conclude the lack of difference in PPM requirements, and 30-day and 1-year mortality between the two cohorts.

**Conclusion:**

TAVI gives satisfactory outcomes for treating stenotic BAV and should be considered clinically.

**Table:** 

Abbreviations, acronyms & symbols	
**AKI**	**= Acute kidney injury**
**AS**	**= Aortic stenosis**
**BAV**	**= Bicuspid aortic valve**
**CI**	**= Confidence interval**
**PPM**	**= Permanent pacemaker**
**PRISMA**	**= Preferred Reporting Items for Systematic Reviews and Meta-Analyses**
**RR**	**= Risk ratio**
**SPSS**	**= Statistical Package for the Social Sciences**
**TAVI**	**= Transcatheter aortic valve implantation**
**TAV**	**= Tricuspid aortic valve**
**TSA**	**= Trial sequential analysis**
**WMD**	**= Weighted mean differences**

## INTRODUCTION

Transcatheter aortic valve implantation (TAVI) is a well-established treatment strategy in patients with severe symptomatic aortic stenosis with high surgical risk for conventional aortic valve replacement^[1,2]^. However, this recommendation was based on clinical trials that excluded patients with bicuspid aortic valve (BAV)^[1,2]^, a common cardiac anomaly present in 0.5-2% of the general population and associated with the development of aortic stenosis (AS) requiring intervention^[3]^. Generally, patients with BAV have larger annular dimensions, may have variable coronary anatomy, more calcified, bulky and irregular aortic valve leaflets, and altered aortic geometry and blood flow^[4,5]^. These differences can complicate the accurate device delivery and apposition of the prosthetic valve during TAVI^[6-8]^. However, outcomes of TAVI in patients with BAV using new-generation valves have shown promising results, with less paravalvular leak and better postprocedural outcomes than early-generation valves^[9-11]^. A significant number of centres around the world have also started performing TAVI on stenotic BAV patients. This systematic review and meta-analysis thus sought to thoroughly examine the literature to compare the outcomes of using TAVI in BAV replacement.

## METHODS

This systematic review and meta-analysis was reported according to the Preferred Reporting Items for Systematic Reviews and Meta-Analyses (PRISMA) statement, and was conducted according to The Cochrane Handbook for Systematic Reviews of Interventions^[12,13]^. Electronic searches were performed on PubMed, Scopus, MEDLINE, and EMBASE from their inception up till October 2019 to identify all publications that reported the use of TAVI in patients with BAV. ClinicalTrials.gov was also searched to identify ongoing or unpublished clinical trials. The search string used was "TAVI" OR "valve implantation" OR "percutaneous" AND "bicuspid valve" OR "bicuspid aorta" OR "bicuspid aortic valve" OR "aortic stenosis". Reference lists of identified papers were searched manually to identify other eligible studies. 

### Inclusion and Exclusion Criteria

Only studies written in English comparing TAVI in at least five patients with stenotic BAV and tricuspid aortic valve (TAV) were included. Non-comparative studies, studies with less than five patients, and studies including re-do valve-in-valve, tricuspid valve or aortic regurgitation were excluded. Articles were screened by three reviewers (JSKC, PE, LHT). All selected articles were systematically assessed with inclusion and exclusion criteria. Conflicts over inclusion were resolved by an independent reviewer (AH). All included studies were critically appraised using the Newcastle-Ottawa Scale. 

### Data Extraction and Reported Outcomes

Summary estimates were manually extracted by three reviewers (SS, PE, LHT). When there were duplicate data, only the most updated data were included. Conflicts over data extraction were resolved by an independent reviewer (JSKC). Primary endpoints included 30-day and 1-year mortality. Secondary endpoints included post-operative stroke, AKI, and need for permanent pacemaker (PPM) implantation. Other baseline, operative and post-operative characteristics were also extracted. 

### Statistical Analysis

Risk ratio (RR [95% confidence interval (CI)]) or weighted mean differences (WMD [95% CI]) were used as summary measures for primary endpoints. Random effects model was used with the Mantel-Haenszel test or inverse variance analysis, as appropriate. Heterogeneity was assessed by the chi-square test and the I^2^ statistic, for which values >0.40 were considered to imply significant heterogeneity. Sensitivity analysis was performed by removing studies individually from the analysis. 

Trial sequential analysis (TSA) was performed on all outcomes using a combination of sample size and event size. O'Brien-Fleming α-spending function was used to adjust the Z-score threshold. Studies with 0 events were handled by adding a constant (1) to both the intervention and control arm. Required information size was estimated from all included studies reporting the analysed variables and incidences calculated from included patients, with a permissible two-sided type 1 error of 5% and type 2 error of 20%. TSA was performed using the Copenhagen trial unit TSA software version 0.9.5.10 beta. 

All P-values were 2-sided, with P<0.05 considered significant. Statistical analyses were performed using Review Manager V.5.3 (Copenhagen: The Nordic Cochrane Centre, The Cochrane Collaboration, 2014) and SPSS software version 25.0 (IBM Corp, Armonk, New York, USA).

## RESULTS

**Fig. 1:**
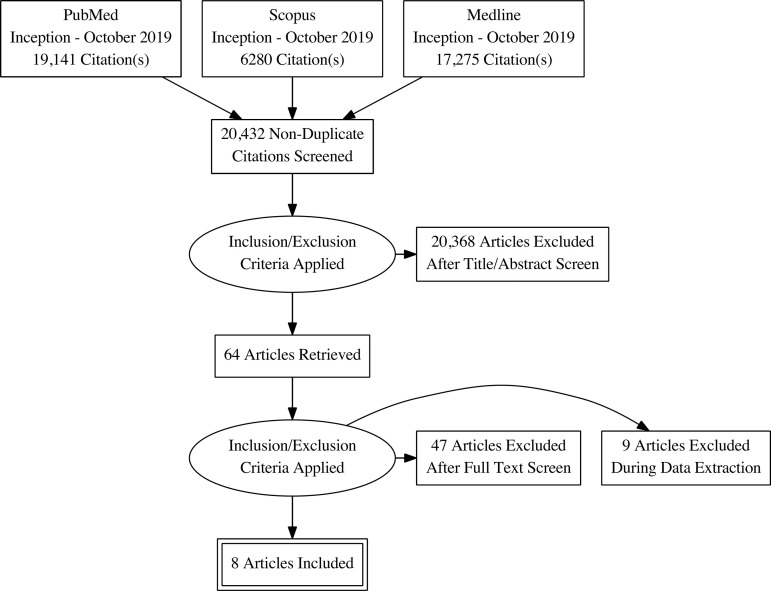
Preferred reporting items for systematic reviews (PRISMA) diagram.

Eight studies were deemed eligible for inclusion in this meta-analysis (Figure 1)^[14-21]^. The studies were excluded due to the lack of reports on TAV and BAV cohorts in the same article, in case series with less than 5 patients or in single cohort studies. The articles represented a total of 3996 patients (917 with BAV and 3079 with TAV, Table 1). An electronic search on ClinicalTrials.gov also identified one relevant single-blinded randomized controlled trial. The study is expected to be completed in 2023 (ClinicalTrials.gov identifier: NCT02541877). Results of the critical appraisal by the Newcastle-Ottawa Scale were summarized in Table 2. Each asterisk represents 1 point, with ≥7 out of 9 points unlikely to have a significant risk of bias. The assessment results showed that all studies were unlikely to have a significant risk of bias. 

**Table 1:** Summary of all included studies.

Article	Year of publication	Study type	BAV (n=1077)	TAV (n=4165)	Summary of article
Aalei-Andabili et al.^[14]^	2018	Retrospective cohort	32	96	TAVI in patients with BAV provides both comparable immediate and mid-term outcomes with TAV and is feasible.
Bauer et al.^[15]^	2014	Prospective cohort	38	1357	In selected patients, TAVI for BAV can provide satisfactory results. Risk of relevant AR appears greater in patients with BAV; however, both 30-day and 1-year mortalities were not elevated in relation to TAV.
De Biase et al.^[16]^	2018	Prospective cohort	83	166	More complex anatomy associated with BAV at baseline leads to lower device success rates, but this is not associated with higher 30-day mortalities.
Kochman et al.^[17]^	2014	Prospective cohort	28	84	Selected high-risk BAV patients can be successfully treated with TAVI and have similar outcomes to non-BAV patients.
Liao et al.^[18]^	2018	Prospective cohort	87	70	TAVI for BAV looks safe and effective, with comparable bioprosthetic valve functionality compared to TAV.
Liu et al.^[19]^	2015	Prospective cohort	15	25	There was no difference between the success of device, 30-day mortality or the 30-day combined endpoints between TAVI in BAV and TAV.
Sannino et al.^[20]^	2017	Retrospective cohort	88	735	TAVI appears safe and effective in BAV, with no differences in post-procedure mortality, or 30-day cardiovascular mortality compared to patients with TAV.
Yoon et al.^[21]^	2017	Prospective and retrospective cohort	546	546	TAVI in BAV was associated with similar prognosis but had lower device success. Procedural differences occurred with early devices, but not with new generation ones.

AR=aortic regurgitation; BAV=bicuspid aortic valve; TAV=tricuspid aortic valve; TAVI=transcatheter aortic valve. implantation.

**Table 2:** Newcastle-Ottawa Scale.

Author	Selection				Comparability		Outcome			
	Representation of patients with bicuspid aortic valve	Selection of patients with tricuspid aortic valve	Ascertainment of exposure	Demonstration that the outcome of interest was not present at the start of the study	Study controls for patient age =✹	Study controls for preoperative cardiac function and cardiovascular co-morbidities =✹	Assessment of outcomes	Follow-up long enough for outcomes to occur	Adequacy of follow-up of cohorts	Overall quality score (maximum=9)
Aalaei-Andabili et al.^[14]^	✹	✹	✹	✹	✹✹		✹		✹	8
Bauer et al.^[15]^	✹	✹	✹	✹	✹✹		✹		✹	8
De Biase et al.^[16]^	✹	✹	✹	✹	✹✹		✹		✹	8
Kochman et al.^[17]^	✹	✹	✹	✹	✹✹		✹	✹	✹	9
Liao et al.^[18]^	✹	✹	✹	✹	✹✹		✹	✹	✹	9
Liu et al.^[19]^	✹	✹	✹	✹	✹✹		✹		✹	8
Sannino et al.^[20]^	✹	✹	✹	✹	✹✹		✹	✹	✹	9
Yoon et al.^[21]^	✹	✹	✹	✹	✹✹		✹	✹	✹	9

The baseline characteristics were summarized in Table 3. The BAV cohort was significantly younger (WMD -0.89 years [-1.60 years, -0.17 year], *P*=0.02), and had a higher trans-aortic valve gradient (WMD 1.73 mmHg [0.31 mmHg, 3.16 mmHg], *P*=0.02), and a larger ascending aortic diameter (WMD 3.92 mm [3.02 mm, 4.83 mm], *P*<0.0001). All other baseline characteristics were not significantly different.

**Table 3:** Baseline characteristics of the included patients. The first number is the number of patients with the event or the sample mean value ± standard deviation of the variable, and the second is the number of patients for whom the event was described. Statistically significant differences were marked with an asterisk (*).

Baseline variable	BAV (n=917)	TAV (n=3079)	OR/WMD [95% CI]	*P*-value
Age (years±SD)	76.8±7.43	78.5±7.12	WMD -0.89 [-1.60, -0.17]	0.02*
Male (%)	562/917 (61.3)	1554/3079 (50.5)	OR 1.10 [0.93, 1.31]	0.27
Diabetes mellitus (%)	226/917 (24.6)	980/3079 (31.8)	OR 1.00 [0.82, 1.21]	0.98
Previous stroke (%)	130/917 (14.2)	357/3079 (11.6)	OR 1.20 [0.94, 1.54]	0.15
Peripheral arterial disease (%)	177/834 (21.2)	690/2913 (23.7)	OR 0.95 [0.67, 1.34]	0.76
Hypertension (%)	603/879 (68.6)	1293/1722 (75.1)	OR 0.95 [0.78, 1.16]	0.61
COPD (%)	194/834 (23.3)	660/2913 (22.7)	OR 1.05 [0.84, 1.32]	0.67
Ischaemic heart disease (%)	175/339 (51.6)	1472/2437 (60.4)	OR 0.99 [0.76, 1.29]	0.92
Previous CABG (%)	75/710 (10.6)	340/2178 (15.6)	OR 0.85 [0.62, 1.17]	0.33
Previous PCI (%)	180/797 (22.6)	709/2248 (31.5)	OR 0.89 [0.71, 1.11]	0.31
NYHA class III-IV (%)	632/797 (79.3)	1874/2248 (83.4)	OR 1.09 [0.86, 1.37]	0.48
Atrial fibrillation (%)	49/273 (17.9)	189/996 (19.0)	OR 0.94 [0.64, 1.38]	0.74
EuroSCORE (%±SD)	17.4±10.5	19.4±12.6	WMD -1.15 [-2.63, 0.33]	0.13
STS score (%±SD)	6.10±3.88	6.53±3.98	WMD 0.06 [-0.28, 0.40]	0.73
Aortic valve area (cm^2^±SD)	0.629±0.173	0.660±0.239	WMD -0.03 [-0.06, 0.01]	0.11
Trans-aortic valve gradient (mmHg±SD)	52.6±17.3	49.8±16.1	WMD 1.73 [0.31, 3.16]	0.02*
Aortic annular diameter (mm±SD)	23.6±4.42	24.3±3.72	WMD -0.23 [-1.63, 1.17]	0.74
Left ventricular ejection fraction (%±SD)	51.3±14.2	53.6±13.0	WMD -2.60 [-5.57, 0.37]	0.09
Ascending aortic diameter (mm±SD)	38.4±5.00	34.2±3.63	WMD 3.92 [3.02, 4.83]	<0.0001*

BAV=bicuspid aortic valve; CABG=coronary artery bypass graft; CI=confidence interval; COPD=chronic obstructive pulmonary disease; NYHA=New York Heart Association; PCI=percutaneous coronary intervention; STS=Society of Thoracic Surgeons; WMD=weighted mean difference

**Fig. 2:**
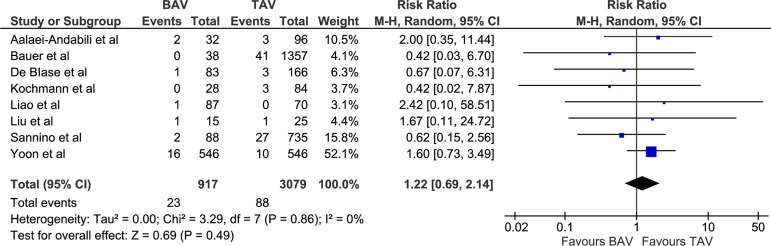
Forest plot for postoperative stroke. BAV=bicuspid aortic valve; CI=confidence interval; M-H=Mantel-Haenszel; TAV=tricuspid aortic valve.

**Fig. 3:**
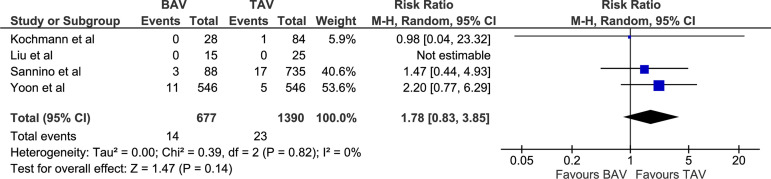
Forest plot for postoperative acute kidney injury. BAV=bicuspid aortic valve; CI=confidence interval; M-H=Mantel-Haenszel; TAV=tricuspid aortic valve

**Fig. 4:**
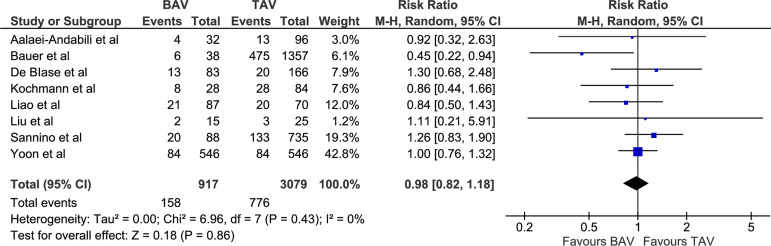
Forest plot for postoperative permanent pacemaker requirement. BAV=bicuspid aortic valve; CI=confidence interval; M-H=Mantel-Haenszel; TAV=tricuspid aortic valve

**Fig. 5:**
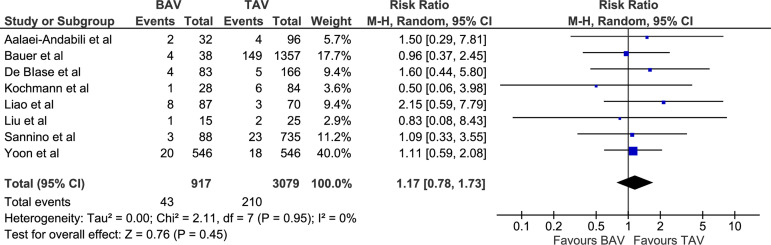
Forest plot for postoperative 30-day mortality. BAV=bicuspid aortic valve; CI=confidence interval; M-H=Mantel-Haenszel; TAV=tricuspid aortic valve

**Fig. 6:**
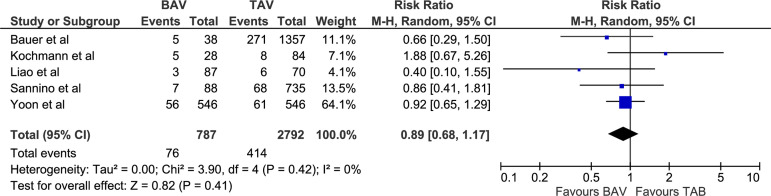
Forest plot for postoperative 1-year mortality. BAV=bicuspid aortic valve; CI=confidence interval; M-H=Mantel-Haenszel; TAV=tricuspid aortic valve

Operative outcomes were summarized in Table 4 and postoperative outcomes in Table 5. All primary and secondary outcomes were not significantly different and did not have significantly heterogeneous data. These included postoperative stroke (RR 1.22 [0.69, 2.14], *P*=0.49; I^2^=0, chi-square=3.29, *P*=0.86; Figure 2), AKI (RR 1.78 [0.83, 3.85], *P*=0.14; I^2^=0, chi-square=0.39, *P*=0.82; Figure 3), PPM requirement (RR 0.98 [0.82, 1.18], *P*=0.86; I^2^=0, chi-square=6.96, *P*=0.43; Figure 4), 30-day mortality (RR 1.17 [0.78, 1.73], *P*=0.44; I^2^=0, chi-square=2.11, *P*=0.95; Figure 5), and 1-year mortality (RR 0.89 [0.68, 1.17], *P*=0.41; I^2^=0, chi-square=3.90, *P*=0.42; Figure 6). However, the BAV cohort had considerable higher rates of significant aortic regurgitation (more than grade 2) postoperatively (RR 1.53 [1.17, 1.99], *P*=0.002). All other operative and postoperative outcomes were not significantly different. Sensitivity analysis revealed that the individual removal of studies from the analysis would not affect the statistical significance of the meta-analytical results of any variable.

**Table 4:** Operative outcomes of the included patients. The first number is the number of patients with the event or the sample mean value ± standard deviation of the variable, and the second is the number of patients for whom the event was described. Statistically significant differences were marked with an asterisk (*).

		BAV (n=917)	TAV (n=3079)	OR [95% CI]	*P*-value
Vascular access route	Transfemoral (%)	689/829 (83.1)	2015/2330 (86.5)	0.82 [0.52, 1.28]	0.37
	Transapical (%)	22/186 (11.8)	216/2272 (9.51)	1.11 [0.68, 1.80]	0.68
	Transaortic (%)	6/284 (2.11)	48/2463 (1.95)	1.14 [0.42, 3.08]	0.80
	Others (%)	4/201 (1.99)	57/2297 (2.48)	0.91 [0.33, 2.47]	0.85
Type of valve used	Sapien XT (%)x	175/606 (28.9)	218/726 (30.0)	1.01 [0.79, 1.29]	0.95
	CoreValve (%)	256/746 (34.3)	1391/2178 (63.9)	1.06 [0.62, 1.83]	0.82
	Venus A (%)	64/102 (62.8)	60/95 (63.2)	0.81 [0.36, 1.83]	0.61
	SAPIEN 3 (%)	232/699 (33.2)	507/2165 (23.4)	1.38 [0.76, 2.53]	0.29
	Lotus (%)	46/629 (7.31)	51/712 (7.16)	0.94 [0.62, 1.43]	0.78
	EvolutR (%)	39/629 (6.20)	114/712 (16.4)	0.49 [0.06, 4.13]	0.51

BAV=bicuspid aortic valve; OR=odds ratio; TAV=tricuspid aortic valve

**Table 5:** Postoperative outcomes of the included patients. The first number is the number of patients with the event or the sample mean value ± standard deviation of the variable, and the second is the number of patients for whom the event was described. Statistically significant differences were marked with an asterisk (*).

	BAV (n=917)	TAV (n=3079)	RR/WMD [95% CI]	*P*-value
Device success (%)	712/830 (85.8)	2812/3009 (93.5)	RR 0.96 [0.90, 1.03]	0.24
Bleeding (%)	83/764 (10.9)	183/1460 (12.5)	RR 1.00 [0.72, 1.39]	0.98
Conversion to open surgery (%)	12/746 (1.61)	16/2178 (0.73)	RR 2.89 [0.53, 15.83]	0.22
Vascular complications (%)	38/802 (4.74)	105/2817 (3.73)	RR 1.02 [0.66, 1.56]	0.94
Trans-aortic valve gradient (mmHg±SD)	9.74±5.96	9.78±5.15	WMD 0.09 [-0.36, 0.54]	0.70
AR more than grade 2 (%)	88/815 (10.8)	303/2984 (10.2)	RR 1.53 [1.17, 1.99]	0.002*
Stroke (%)	23/917 (2.51)	88/3179 (2.77)	RR 1.22 [0.69, 2.14]	0.49
Acute kidney injury (%)	14/677 (2.07)	23/1390 (1.66)	RR 1.78 [0.83, 3.85]	0.14
PPM requirement (%)	158/917 (17.2)	776/3079 (25.2)	RR 0.98 [0.82, 1.18]	0.86
30-day mortality (%)	43/917 (4.69)	210/3079 (6.82)	RR 1.17 [0.78, 1.73]	0.45
1-year mortality (%)	76/787 (9.66)	414/2792 (14.8)	RR 0.89 [0.68, 1.17]	0.41

AR=aortic regurgitation; BAV=bicuspid aortic valve; OR=odds ratio; PPM=permanent pacemaker; RR=risk ratio; SD=standard deviation; TAV=tricuspid aortic valve; WMD=weighted mean difference

**Fig. 7:**
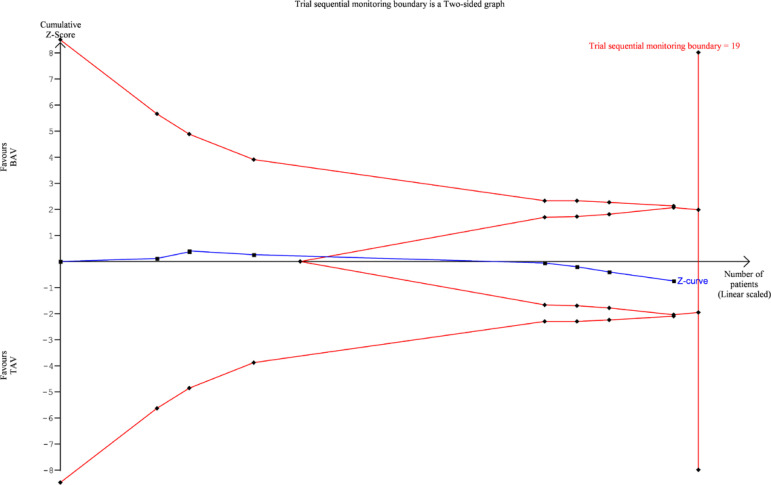
Trial sequential analysis diagram for 30-day mortality. BAV=bicuspid aortic valve; TAV=tricuspid aortic valve

**Fig. 8:**
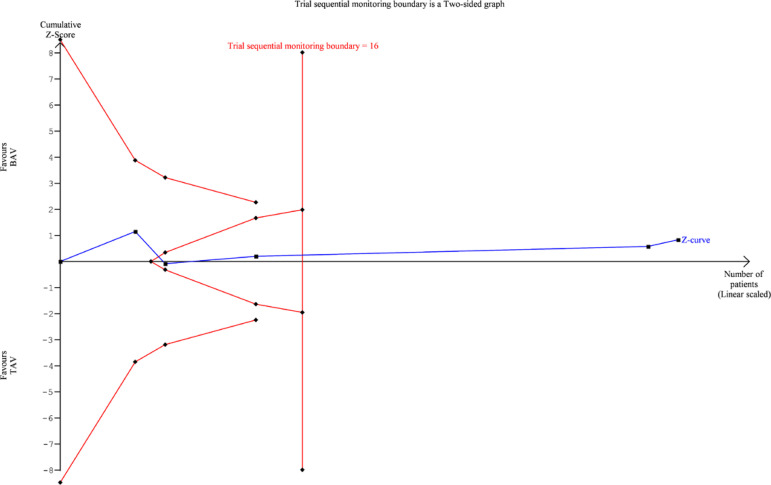
Trial sequential analysis diagram for 1-year mortality. BAV=bicuspid aortic valve; TAV=tricuspid aortic valve

**Fig. 9:**
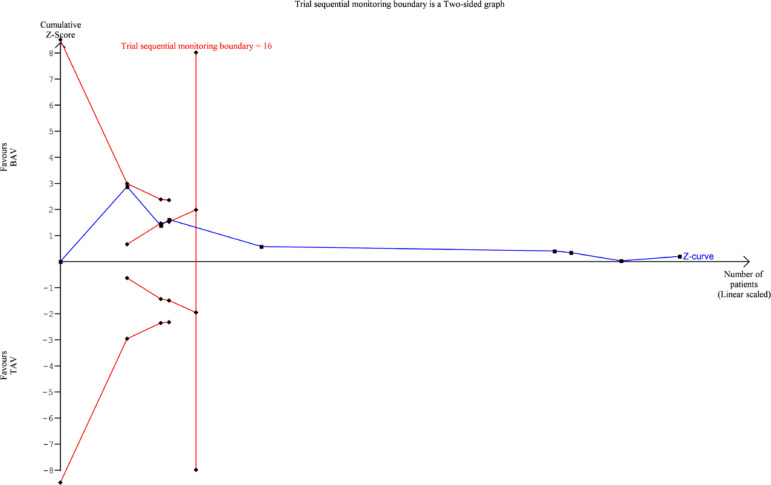
Trial sequential analysis diagram for postoperative permanent pacemaker requirement. BAV=bicuspid aortic valve; TAV=tricuspid aortic valve

**Fig. 10:**
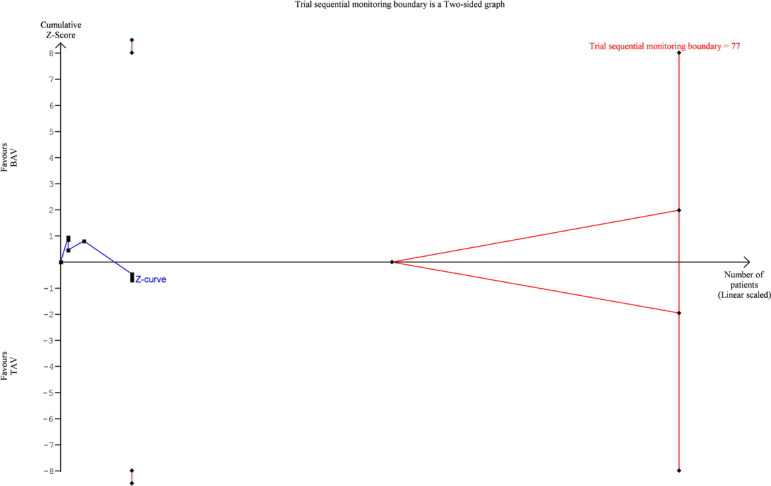
Trial sequential analysis diagram for postoperative stroke. BAV=bicuspid aortic valve; TAV=tricuspid aortic valve

TSA was performed for 30-day mortality, 1-year mortality, PPM requirement, and postoperative stroke. For 30-day mortality (Figure 7), 1-year mortality (Figure 8) and PPM requirement (Figure 9), the cumulative Z curve of the study data crossed the futility boundary and/or the trial sequential monitoring boundary, indicating that the meta-analysis of these variables was conclusive. However, the cumulative Z curves for postoperative stroke (Figure 10) did not cross any of the boundaries, indicating that meta-analyses of these variables were inconclusive, and further studies were required. TSA was not possible for AKI due to the small sample and event sizes.

## DISCUSSION

TAVI has not been fully explored in patients with BAV, and the latest guidelines did not fully support the use of TAVI in BAV patients^[22,23]^. We thus evaluated the existing evidence for the safety of TAVI in stenotic BAV compared to that in TAV. In this study, a total of eight articles with 917 stenotic BAV and 3079 stenotic TAV patients were meta-analysed. We showed no significant difference in primary and secondary outcomes, including AKI, PPM requirement, stroke, and 30-day mortality. TSA results confirmed that 30-day and 1-year mortality, as well as PPM requirements, were not significantly different between BAV and TAV, while more evidence was required for stroke. As such, TAVI is largely safe for clinical use in stenotic BAV patients.

**Fig. 11:**
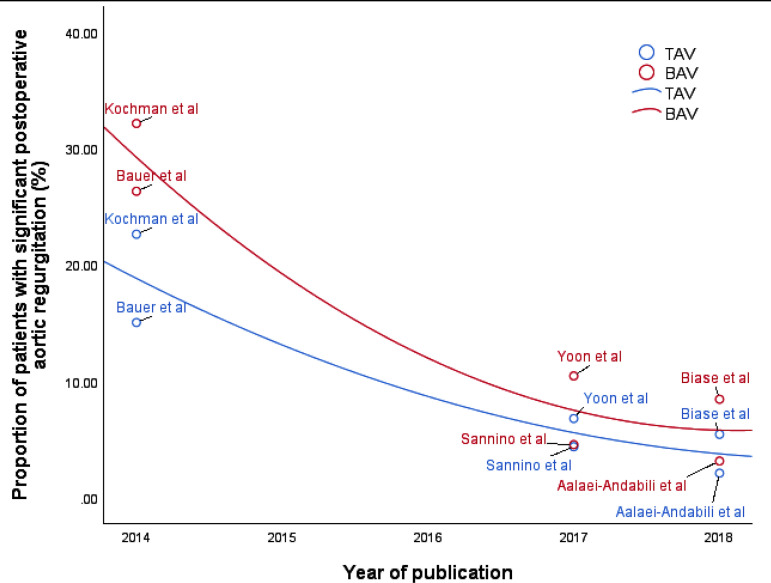
Rate of significant postoperative aortic regurgitation in studies according to the year of publication. A decreasing trend is apparent. BAV=bicuspid aortic valve; TAV=tricuspid aortic valve

However, BAV was associated with a considerable higher rate of significant aortic regurgitation postoperatively (*P*=0.002). It should be noted that if studies that started recruitment before 2012 were excluded, i.e. Bauer et al.^[15]^, Kochman et al.^[17]^ and Yoon et al.^[21]^, the difference in the rates of significant postoperative aortic regurgitation would become statistically insignificant (RR 1.31 [0.67, 2.54], *P*=0.43; I^2^=0, chi-square=0.33, *P*=0.85). This reflected that there was a learning curve for TAVI and the inclusion of older, earlier procedures skewed the data-in fact, Yoon et al.^[21]^ included procedures performed as early as 2005. A plot between the year of publication and the rate of significant postoperative aortic regurgitation showed a decreasing trend across the years (Figure 11). The idea of a learning curve for TAVI has been suggested by others and, therefore, having contemporary data is important^[24,25]^.

Previous meta-analyses on the same topic were published by Takagi et al.^[26]^ and Ueshima et al.^[27]^. Our results largely agree with these studies. Nonetheless, to the best of the authors' knowledge, this was the first meta-analysis to utilize TSA in evaluating TAVI in BAV *versus* TAV^[28]^. TSA builds on the simple fact that the conclusiveness of any evidence increases with the sample size studied, and that the stronger a true effect is, the fewer the number of subjects that need to be studied for the observed effects to be conclusive. TSA results are presented as plots: whenever the Z-score curve (blue line) crosses either the statistical significance boundary (outer oblique boundaries), the futility boundary (inner oblique boundaries), or the trial sequential monitoring boundary (vertical boundary), the results may be considered stable and conclusive. Our results justified further studies designed to evaluate postoperative stroke with adequate power. In fact, with almost 4000 patients included in comparative studies, TSA should be encouraged in subsequent meta-analyses to check if a definitive conclusion has been reached.

This meta-analysis had several limitations. First, it was possible that the manual electronic search had lost articles eligible for inclusion in this study, as well as studies written in non-English languages. Second, late mortality (>1-year follow-up) could not be analysed since only 1 study (Yoon et al.^[21]^ followed patients for more than 1 year with adequate data for analysis. These might mean that long-term data of TAVI in TAV were likely not extrapolatable to BAV patients. This is an important consideration for future studies.

Third, subgroup analysis by the BAV subtype was not possible, as none of the included studies reported separate data by BAV subtypes. This has been considered a key parameter that should be assessed in preoperative imaging of BAV patients considered for TAVI. Similarly, subgroup analysis by the type of valve used was not possible due to studies including a mixture of different valves, often from different generations. Reporting outcomes by the type of valve could give a better understanding of whether the benefits of newer generation valves in TAV were applicable in BAV. These should be considered in future studies.

## CONCLUSION

The use of TAVI provides satisfactory outcomes in stenotic BAV patients, largely comparable to those in stenotic TAV patients. Given the current literature, TAVI may be considered clinically for patients with stenotic BAV.

**Table:** 

Authors' roles & responsibilities	
JSKC	Substantial contributions to the conception or design of the work; or the acquisition, analysis, or interpretation of data for the work; drafting the work or revising it critically for important intellectual content; final approval of the version to be published
SS	Substantial contributions to the conception or design of the work; or the acquisition, analysis, or interpretation of data for the work; drafting the work or revising it critically for important intellectual content; final approval of the version to be published
PE	Substantial contributions to the conception or design of the work; or the acquisition, analysis, or interpretation of data for the work; drafting the work or revising it critically for important intellectual content; final approval of the version to be published
LHT	Substantial contributions to the conception or design of the work; or the acquisition, analysis, or interpretation of data for the work; drafting the work or revising it critically for important intellectual content; final approval of the version to be published
AH	Substantial contributions to the conception or design of the work; or the acquisition, analysis, or interpretation of data for the work; drafting the work or revising it critically for important intellectual content; final approval of the version to be published

## Supplementary Material

- Cardiovascular Evidence Review Collaboration (CvERC) investigators
- Jeffrey Shi Kai Chan, Division of Cardiology, Department of Medicine and Therapeutics, Prince of Wales Hospital, Hong Kong; Faculty of Medicine, The Chinese University of Hong Kong, Hong Kong
- Amer Harky, Department of Cardiothoracic Surgery, Liverpool Heart and Chest Hospital, Liverpool, United Kingdom
- Hummad Anwar, University Hospitals of Leicester NHS Trust, Leicester, United Kingdom
- Christiana Bithas, Southport and Ormskirk Hospital NHS Trust, Southport, United Kingdom
- Grace Chaplin, School of Medicine, University of Liverpool, Liverpool, United Kingdom
- Chun Ming Chiu, Brighton and Sussex Medical School, University of Sussex, East Sussex, United Kingdom
- Fiona Choi, School of Medicine, University of Liverpool, Liverpool, United Kingdom
- Dileep Duwa, Department of Cardiothoracic Surgery, Liverpool Heart and Chest Hospital, Liverpool, United Kingdom
- Ka Siu Fan, St George's Medical School, University of London, United Kingdom
- Shahi Ghani, St George's University of London, Tooting, United Kingdom
- Ciarin Grafton-Clarke, School of Life Sciences, University of Leicester, Leicester, United Kingdom
- Shubhi Gupta, School of Medicine, University of Liverpool, Liverpool, United Kingdom
- Kantida Koysombat, School of Medicine, University of Liverpool, Liverpool, United Kingdom
- Ji Yen Ku, School of Medicine, University of Liverpool, Liverpool, United Kingdom
- Ter-Er Kusu-Orkar, Pilgrim Hospital, Boston, United Kingdom
- Jason Lee, School of Medicine, University of Liverpool, Liverpool, United Kingdom
- Beverly MacCarthy-Ofosu, Countess of Chester Hospital, Chester, United Kingdom
- Nina Oguamanam, East Sussex NHS Trust, East Sussex, United Kingdom
- Pratik Rai, School of Medicine, University of Liverpool, Liverpool, United Kingdom
- Siddiq Ur Rehman, Southport and Ormskirk Hospital NHS Trust, Southport, United Kingdom
- Sugeevan Savarimuthu, Broomfield Hospital, Chelmsford, East of England Deanery, United Kingdom
- Sukhdeep Singh, Faculty of Medicine, The Chinese University of Hong Kong, Hong Kong
- Pawel Sokal, School of Medicine, University of Liverpool, Liverpool, United Kingdom
- Wing Yan Elizabeth Wong, Brighton and Sussex Medical School, University of Sussex, East Sussex, United Kingdom
- Sevim Zaim, School of Medicine, University of Liverpool, Liverpool, United Kingdom
